# ﻿*Panaxsiamensis* J. Wen, a new species of the ginseng genus (*Panax*, Araliaceae) from northern Thailand

**DOI:** 10.3897/phytokeys.234.106289

**Published:** 2023-10-06

**Authors:** Jun Wen, Gary Krupnick, Hans-Joachim Esser

**Affiliations:** 1 Department of Botany, National Museum of Natural History, Smithsonian Institution, PO Box 37012, Washington, DC 20013-7012, USA Department of Botany, National Museum of Natural History, Smithsonian Institution Washington United States of America; 2 Botanische Staatssammlung München, Staatliche Naturwissenschaftliche Sammlungen Bayerns (SNSB), Menzinger Straße 67, 80638 München, Germany Botanische Staatssammlung München, Staatliche Naturwissenschaftliche Sammlungen Bayerns (SNSB) München Germany

**Keywords:** Araliaceae, conservation, ginseng genus, *Panax*, *Panaxsiamensis* J. Wen

## Abstract

We herein describe a new species, *Panaxsiamensis* J. Wen, from the tropical monsoon forests in northern Thailand. *Panaxsiamensis* is characterized by a combination of characters including horizontally elongated rhizomes with thick internodes, 3–5 whorled leaves each with 7–9 sessile and lanceolate leaflets, lanceolate bracteoles not persisting at the fruiting stage, 2-locular ovaries, and red fruits with a black top. The new species is most closely related to *Panaxzingiberensis* C.Y. Wu & Feng from southeastern Yunnan province of China, sharing the character of sessile leaflets, but differing in that *P.siamensis* has well developed, elongated rhizomes (vs. compact, ginger-like rhizomes and rootstock in *P.zingiberensis*), and 7–9 leaflets (vs. (3–) 5–7 leaflets in *P.zingiberensis*). We also compare *Panaxsiamensis* to other related Asian *Panax* species, including *P.assamicus* Banerjee, *P.bipinnatifidus* Seem., *P.pseudoginseng* Wallich, and *P.vietnamensis* Ha & Grushv. The new taxon is preliminarily assessed as Vulnerable (VU D2), according to the IUCN Red List criteria. A taxonomic key is provided to facilitate the identification of *P.siamensis* and its close allies.

## ﻿Introduction

*Panax* L., the ginseng genus, is an economically important lineage with several medicinally significant species, e.g., *Panaxginseng* C.A.Meyer (ginseng), *P.quinquefolius* L. (American ginseng), *P.notoginseng* (Burkill) F. H. Chen ex C. Y. Wu et al. (sanchi), *P.zingiberensis* C.Y. Wu & Feng (ginger-like sanchi), and *P.vietnamensis* Ha & Grushv. (Vietnamese ginseng) (Zhou et al. 1975; Ha and Grushvitzky 1985; Wen and Zimmer 1996). The genus consists of c. 18 species disjunctly distributed in eastern Asia to the Himalayas and eastern North America, showing a classical eastern Asian – eastern North American biogeographic disjunction (Wen and Zimmer 1996; Wen 1999, 2001; Lee and Wen 2004; Zuo et al. 2015). It is one of the c. 50 genera of the ginseng family Araliaceae (Wen et al. 2001; Plunkett et al. 2018; Gallego‐Narbón et al. 2022).

In spite of its economic (Hu 1976; Proctor 1996) and biogeographic (Wen 1999; Zuo et al. 2017) importance, the species delimitation of Asian *Panax* has been controversial, largely involving the circumscription of *Panaxjaponicus* C.A. Meyer and *Panaxpseudoginseng* Wallich (Li 1942; Hara 1966, 1971; Zhou et al. 1975; Hoo and Tseng 1978; Yang 1981; Xiang and Lowry 2007). Molecular phylogenetic analyses have strongly suggested that *Panaxjaponicus* is endemic to Japan and forms a clade with two of the medicinally important species: *Panaxginseng* and *P.quinquefolius* (Wen and Zimmer 1996; Lee and Wen 2004; Zuo et al. 2011, 2017). A number of studies have supported the distinctiveness of *Panaxpseudoginseng* as a species that is narrowly distributed in Nepal and adjacent areas of Xizang, China, and as closely related to *Panaxstipuleanatus* Tsai & Feng, which is from southwestern China and northern Vietnam (Lee and Wen 2004; Zuo et al. 2011, 2017). Zhou et al. (1975) also described *Panaxzingiberensis* from southeast and southern Yunnan that has ginger-like roots persistent in older plants, and leaflets without petiolules.

During our revisionary studies of *Panax*, we discovered a new species from northern Thailand. This species had been recognized as Araliapseudoginseng(Wallich)Benth. ex C. B. Clarkevar.angustifolia (Burkill) Craib (Craib 1931). Hara (1971) and more recently Esser and Jebb (2019) treated it in the genus *Panax* [as P.pseudoginsengWallichvar.angustifolius (Burkill) H. L. Li], which has been treated as *Panaxbipinnatifidus* Seem. (Zuo et al. 2015, 2017). The new species is highly distinct from *Panaxbipinnatifidus* and several related species from Asia (see Discussion below).

## ﻿Material and methods

Descriptions and measurements of morphological characters of the new species were based on field observations of living plants and herbarium specimens at A, BKF, CMUB, E, K and US. For comparative studies with other *Panax* species, we examined herbarium specimens from the following herbaria: A, ABD, ASSAM, B, BKF, BM, BSHC, C, CAL, CAS, CMUB, E, GH, IBSC, K, KATH, K-W, KUN, L, LBG, LE, MO, NBU, NY, P, PE, PH, TCD, TI, U, UC, US, W, WH, and WU (abbreviations following Thiers 2020). We also examined images of type specimens and other herbarium specimens on JSTOR Global Plants (http://plants.jstor.org), the Chinese Virtual Herbarium Website (http://www.cvh.ac.cn/), and National Specimen Information Infrastructure (http://www.nsii.org.cn/).

## ﻿Taxonomic treatment

### ﻿ Panax siamensis

Taxon classificationPlantaeApialesAraliaceae

J. Wen sp. nov.

2929F89C-CFCA-5A0A-B934-3644B30263CB

urn:lsid:ipni.org:names:77328210-1

Figs 1 , 2

#### Type.

Thailand. Chiang Mai. A southerly ridge of Doi Pa Mawn, an easterly spur of Doi Angka (i.e., Doi Inthanon), ca. 1350 m, berries bright red with a black top, 30 Dec 1926, in fr, *H. B. G. Garrett 364* (holotype: BKF!; isotypes: ABD!, C!, E!, K!, TCD!).

**Figure 1.**
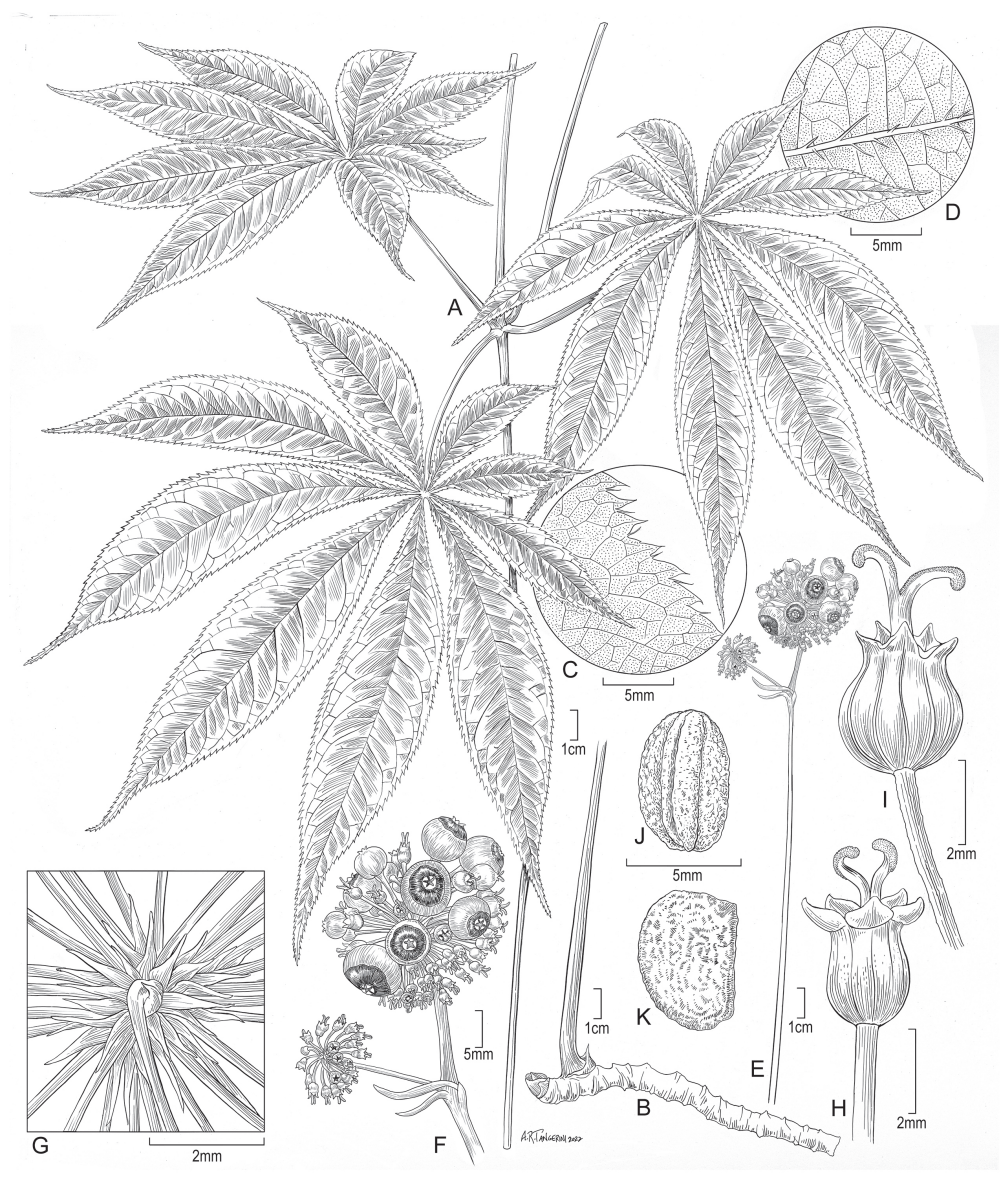
Illustration of *Panaxsiamensis* J. Wen based on *J. Wen 7371* (US) **A** habit **B** horizontal rhizome with base of stem **C** leaflet margin **D** leaflet abaxial surface **E** infructescence **F** enlarged infructescence **G** bracteoles subtending pedicels at flowering stage **H** older flowers after petals falling off, showing 2-locular ovary **I** young fruiting stage **J** seed **K** seed surface.

#### Diagnosis.

Rhizomes horizontally elongated with thick internodes. Leaves 3—5 at the tip of stem, exstipulate, with 7–9 leaflets; leaflets sessile or nearly so, tapering toward the base, lanceolate, long acuminate at apex, long acute at base, serrulate to doubly so at margin, membranaceous, bristly along veins and veinlets on both surfaces. Ovary 2-locular. Fruits subglobose, bright red with a black top, persistent stigmas recurved, 1–2 seeded, 5–6 mm long, 7–8 mm wide, 3–3.5 mm thick. Seeds ovate, 5–5.5 mm long, 3–4 mm wide, 2.5–3.5 mm thick, surface rough.

**Figure 2.**
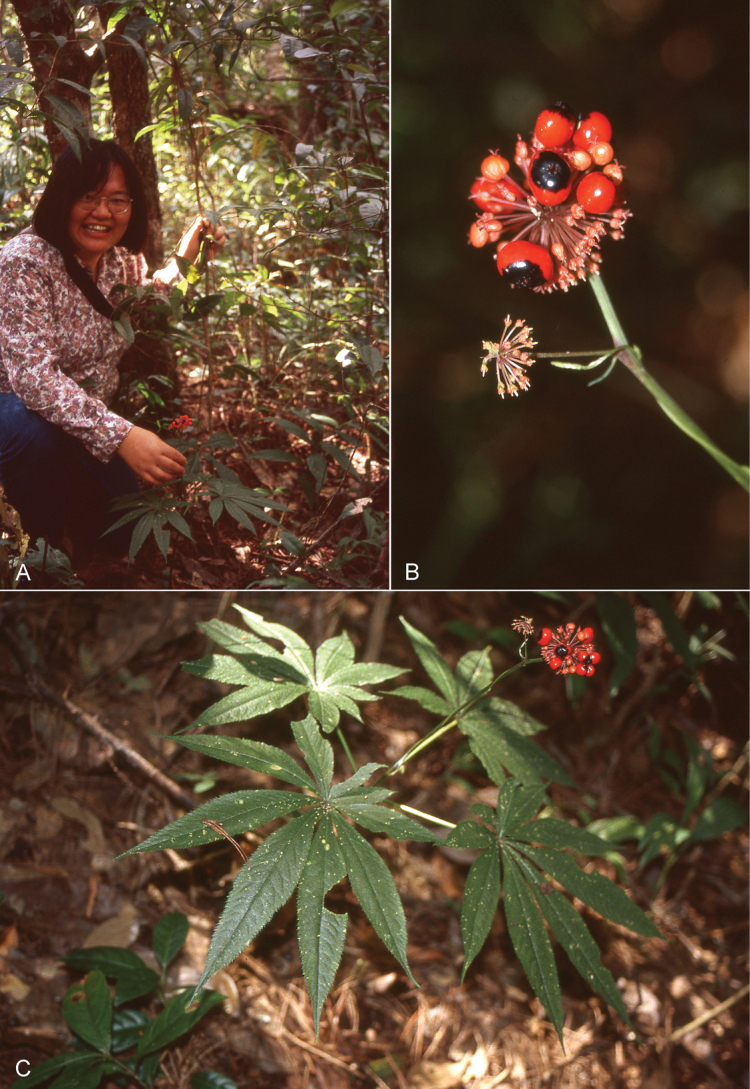
Field images of *Panaxsiamensis* and its habitat **A** habitat in monsoon evergreen forest **B** infructescence showing red fruits with a black top **C** habit. Photo credit: **A** (Ricky Ward), **B, C** (Jun Wen).

#### Description.

Perennial herb, 60–70 cm, hermaphrodite. Rhizomes horizontally elongated with thick internodes, brown outside, whitish inside; stems light green, 30–45 cm. Leaves 3–5 at the tip of the stem, with 7–9 leaflets, exstipulate, petioles 7–11 cm, glabrous; leaflets sessile or nearly so, tapering toward the base, lanceolate, basal leaflets 7–10 cm long, 2–2.5 cm wide, upper leaflets 13–17.5 cm long, 2–3 cm wide, long acuminate at apex, long acute at base, serrulate to doubly so at margin, membranaceous, bristly along veins and veinlets on both surfaces, bristles or setae 1–2.5 mm long, lateral veins 17–25 per side. Inflorescence consisting of a terminal umbel, sometimes with 1–2 lateral umbels, peduncle 15–20 cm, glabrous, terminal umbels with 70–90 flowers, lateral umbels with 20–30 flowers; pedicels puberulent to slightly so, pale light green, with dark violet color at both ends at late flowering to fruiting stage, 10–13 mm in terminal umbels, 6–8 mm in lateral umbels; bracteoles lanceolate, membranaceous, glabrous, 1.3–1.6 mm long, 0.2–0.3 mm wide, mostly not persistent at fruiting stage. Sepals 5, triangular, 0.4–0.5 mm long, 0.6–0.7 mm wide; petals light green, 1.6–2 mm long, 1–1.1 mm wide; anthers white, filaments pale light green, 2–2.2 mm; ovary 2-locular, stigma white. Fruits subglobose, bright red with a black top, persistent stigmas recurved, 1–2 seeded, 5–6 mm long, 7–8 mm wide, 3–3.5 mm thick. Seeds ovate, 5–5.5 mm long, 3–4 mm wide, 2.5–3.5 mm thick, surface rough.

#### Additional specimens examined.

Thailand. Chiang Mai: Mae Soi Ridge, Mae Soi Subdistrict, near Ban Bah Cluary (Meo Village), 1475 m, 11 Aug 1991, in fl, shaded place, mixed evergreen hardwood and pine forest, *J. F. Maxwell 91-722* (A, CMUB, E); Ob Luang National Park, Mae Soi Conservation Area, Bah Gluay (Hmong) village area, between the Ranger Station and the village, on the N side of the road passing through the Bah Gluay village, elev. 1550 m, evergreen forest, plant c. 60–70 m tall, fruits red with a black top, 04 Nov 2003, fr, *J. Wen 7371* (KUN, US).

#### Phenology.

Flowering in August; fruiting in November to December.

#### Distribution.

Northern Thailand; elevation 1350–1550 m.

#### Ecology.

In shaded areas of monsoon evergreen hardwood forests, or in mixed evergreen hardwood and pine forests, granite bedrock.

#### Etymology.

The species epithet denotes the geographic distribution of the species in Thailand.

##### ﻿Conservation

The area of occupancy (AOO) of this species as measured with a 2×2 grid is estimated to be 12 km^2^; the extent of occurrence (EOO) is estimated to be 39.54 km^2^. Apart from habitat destruction, no specific threats are known. It is not found in cultivation, and it is not known from any *ex situ* collections. With a restricted area of occupancy, only three known locations, and a plausible future threat due to stochastic events or habitat disturbance brought about by human activities near villages, *Panaxsiamensis* is preliminarily assessed with a status of Vulnerable (VU D2) according to IUCN Red List Criteria (IUCN 2012, 2022).

## ﻿Discussion

The new species *Panaxsiamensis* is distinguished from other *Panax* species by a combination of characters including horizontally elongated rhizomes with thick internodes, 3–5 whorled leaves each with 7–9 sessile and lanceolate leaflets, lanceolate bracteoles not persisting at the fruiting stage, 2-locular ovaries, and red fruits with a black top. The new taxon is sister to *Panaxzingiberensis* from southeastern Yunnan province of China (J. Wen, unpublished), sharing the character of sessile leaflets. The two species differ in that *P.siamensis* has elongated rhizomes (vs. compact ginger-like rhizomes in *P.zingiberensis*), and 7–9 leaflets (vs. 5–7 leaflets in *P.zingiberensis*). As *P.zingiberensis* is medicinally important (Zhou et al. 1975), it is important to explore the chemistry of *P.siamensis* to test its medicinal value.

*Panaxsiamensis* is similar to *Panaxvietnamensis* from Vietnam and southwestern China in that both species have bamboo-like horizontally elongated rhizomes and 2-locular ovaries. They can be easily differentiated in that *P.vietnamensis* usually has 5 (–7) leaflets (vs. 7–9 leaflets in *P.siamensis*) that are ovate to elliptic (vs. lanceolate in *P.siamensis*), petiolules 8–15 mm long (vs. sessile leaflets without conspicuous petiolules in *P.siamensis*), and glandular pedicels (puberulent pedicels in *P.siamensis*) (Ha and Grushvitzky 1985; Wen 2001; Duy et al. 2016).

Even though *Panaxsiamensis* was recently treated as part of *P.pseudoginseng* (Esser and Jebb 2019), the new species is highly distinct from *Panaxpseudoginseng*, which is narrowly distributed in Nepal and neighboring Xizang of China (Wallich 1829; Wen 2001). *Panaxpseudoginseng* has fusiform tubers that are single or fascicled (vs. tubers absent in *P.siamensis*), rhizomes short and upright (vs. elongate and horizontal in *P.siamensis*), leaves with stipules (stipules absent in *P.siamensis*), and fruits that are red throughout (vs. fruits red with a black top in *P.siamensis*) (Wen 2001; Yoo et al. 2001).

Panaxpseudoginsengvar.angustifolius is now considered as part of *Panaxbipinnatifidus* Seem. (Wen 2001; Zuo et al. 2015). Burkill (1902) originally described Araliaquinquefoliavar.angustifolia Burkill as a taxon from the Sikkim, Bhutan and Khasia Hills (Assam), citing specimens belonging to two species, *P.bipinnatifidus* Seem. and *P.assamicus* R.N. Banerjee. *Panaxsiamensis* resembles *P.assamicus* in the lanceolate leaflets that are sessile or nearly so. They differ in that *P.assamicus* has mostly 5 (–7) leaflets (vs. 7–9 leaflets in *P.siamensis*), persistent bracteoles (vs. bracteoles mostly not persisting in fruiting stage in *P.siamensis*), pilose pedicels (vs. puberulent pedicels with extremely short hair-like structures in *P.siamensis*), oblong sepals (vs. triangular sepals in *P.siamensis*), and 2—3 (–4) locular ovaries (vs. 2-locular ovaries in *P.siamensis*). *Panaxassamicus* is also a much taller herbaceous species that is 70–150 cm tall (vs. *P.siamensis* 60–70 cm tall). Their habitats are also highly distinctive, with *P.siamensis* in tropical monsoon forests and *P.assamicus* in temperate deciduous or mixed forests.

*Panaxsiamensis* can be easily distinguished from *P.bipinnatifidus* in that the latter has horizontal rhizomes with slender internodes and subglobose nodes (vs. horizontal rhizomes with thick and short internodes, i.e., bamboo-like, in *P.siamensis*), leaves with 5 (–7) leaflets (vs. 7–9 leaflets in *P.siamensis*), and terminal umbels with fewer flowers (25–50 flowers in *P.bipinnatifidus*, vs. 70–90 flowers in *P.siamensis*). Ecologically, *Panaxbipinnatifidus* occurs in deciduous or mixed deciduous and coniferous forests in western China to the Himalayas at higher elevations of 2000–3600 m (Wen 2001), while *P.siamensis* occurs in monsoon evergreen hardwood forests, or in mixed evergreen hardwood and pine forests around 1350–1550 m.

We herein provide a key to *Panaxsiamensis* and its close allies to help differentiate the species.

### ﻿Key to *Panaxsiamensis* and its close congeneric allies

**Table:** 

1	Rhizomes elongate and creeping; fruits red with a black top	**2**
–	Rhizomes short and upright; fruits red throughout	**8**
2	Rhizomes with slender internodes and subglobose nodes	***P.bipinnatifidus***
–	Rhizomes with short and thick internodes	**3**
3	Leaflets usually with petiolules	**4**
–	Leaflets usually without petiolules	**6**
4	Leaves subtended by stipules, leaflets usually divided at the margin	***P.stipuleanatus***
–	Leaves without stipules, leaflets usually undivided, only occasionally divided	**5**
5	Leaves with 5 (rarely 3 or 7) leaflets, leaflets oblong or ovate, occasionally lanceolate, light green; ovaries 2-locular	***P.vietnamensis***
–	Leaves with 7–9 (rarely 5) leaflets, leaflets lanceolate to narrowly so, dark green; ovaries 2—4-locular	***P.wangianus***
6	Roots ginger-like, persistent in older plants; leaflets elliptic to obovate	***P.zingiberensis***
–	Roots tuberous, decayed in older plants; leaflets lanceolate	**7**
7	Plant 70–150 cm tall; leaflets usually 5 to occasionally 7; bracteoles subtending pedicels persistent into fruiting stage; ovaries 2—3 (–4) –locular	***P.assamicus***
–	Plant 60—70 cm tall; leaflets 7—9; bracteoles subtending pedicels mostly not persisting at fruiting stage; ovaries 2—locular	***P.siamensis***
8	Roots singly, sometimes forked; stipules linear in shape; each umbel mostly with 80–100 flowers	***P.notoginseng***
–	Roots usually fascicled; stipules ovate in shape; each umbel mostly with 40–65 flowers	***P.pseudoginseng***

## Supplementary Material

XML Treatment for Panax siamensis

## ﻿Additional information

### Author ORCIDs

Jun Wen https://orcid.org/0000-0001-6353-522X

Gary Krupnick https://orcid.org/0000-0002-1357-4826

Hans-Joachim (Hajo) Esser https://orcid.org/0000-0001-6518-8612

### Data availability

All of the data that support the findings of this study are available in the main text or Supplementary Information.

## References

[B1] Burkill IH (1902) Ginseng in China. Bulletin of Miscellaneous Information. Royal Gardens, Kew 1902: 4–11. 10.2307/4114308

[B2] Craib WG (1931) Flora Siamensis Enumeratio. Vol. 1. Bangkok: Siam Society.

[B3] Duy NV Trieu LN Chinh ND Tran VT (2016) A new variety of *Panax* (Araliaceae) from Lam Vien Plateau, Vietnam and Its molecular evidence. Phytotaxa 277(1): 47–58. 10.11646/phytotaxa.277.1.4

[B4] Esser H-J Jebb MHP (2019) Araliaceae. In: Balslev H Chayāmarit K (Eds) Flora of Thailand 14(2).Bangkok: Forest Herbarium, Department of National Parks, Wildlife and Plant Conservation, 185–251.

[B5] Gallego‐Narbón A Wen J Liu J Valcárcel V (2022) Hybridization and genome duplication for early evolutionary success in the Asian Palmate group of Araliaceae. Journal of Systematics and Evolution 60(6): 1303–1318. 10.1111/jse.12906

[B6] Ha DT Grushvitzky IV (1985) A new species of the genus *Panax* (Araliaceae) from Vietnam. Botanicheskii Zhurnal 70: 519–522.

[B7] Hara H (1966) The flora of eastern Himalaya. Tokyo: The University of Tokyo Press.

[B8] Hara H (1971) The flora of eastern Himalaya. Second Report. Tokyo: The University of Tokyo Press.

[B9] Hoo G Tseng CJ (1978) Araliaceae. In: Hoo G Tseng CJ (Eds) Flora Reipublicae Popularis Sinicae. Beijing: Science Press, Beijing.54: 1–190.

[B10] Hu SY (1976) The genus *Panax* (ginseng) in Chinese medicine. Economic Botany 30(1): 11–28. 10.1007/BF02866780

[B11] IUCN (2012) IUCN Red List Categories and Criteria, Version 3.1 (2^nd^ edn.). Gland and Cambridge, 32 pp.

[B12] IUCN (2022) The IUCN Red List of Threatened Species. Version 2022-2. https://www.iucnredlist.org/

[B13] Lee CH Wen J (2004) Phylogeny of *Panax* using chloroplast *trnC-trnD* intergenic region and the utility of *trnC-trnD* in interspecific studies of plants. Molecular Phylogenetics and Evolution 31(3): 894–903. 10.1016/j.ympev.2003.10.009 15120387

[B14] Li HL (1942) The Araliaceae of China. Sargentia 2: 1–134. 10.5962/p.265316

[B15] Plunkett GM Wen J Lowry PP Mitchell AD Henwood MJ Fiaschi P (2018) Araliaceae. In: Kadereit JW Bittrich V (Eds) Flowering Plants. Eudicots. The Families and Genera of Vascular Plants. Berlin: Springer.15: 413–446. 10.1007/978-3-319-93605-5_4

[B16] Proctor JTA (1996) Ginseng: old crop, new directions. In: Janick J (Ed.) Progress in New Crops, Proceedings Third National Symposium, New Crops: new opportunities, new technologies.Alexandria, Virginia: ASHS Press, 565–577.

[B17] Thiers B (2020) Index Herbariorum: A global directory of public herbaria and associated staff. New York Botanical Garden’s virtual Herbarium. http://sweetgum.nybg.org/ih/

[B18] Wallich N (1829) An account of the Nipal ginseng. Transactions of the Medical and Physical Society of Calcutta 4: 115–120.

[B19] Wen J (1999) Evolution of eastern Asian and eastern North American disjunct distributions in flowering plants. Annual Review of Ecology and Systematics 30(1): 421–455. 10.1146/annurev.ecolsys.30.1.421

[B20] Wen J (2001) Species diversity, nomenclature, phylogeny, biogeography, and classification of the ginseng genus (*Panax* L., Araliaceae). In: Punja ZK (Ed.) Proceedings of the International Ginseng Workshop — utilization of biotechnological, genetic & cultural approaches for North American & Asian ginseng improvement.Simon Fraser University, Canada, 67–88.

[B21] Wen J Zimmer EA (1996) Phylogeny and biogeography of *Panax* L. (the ginseng genus, Araliaceae): Inferences from ITS sequences of nuclear ribosomal DNA. Molecular Phylogenetics and Evolution 6(2): 167–177. 10.1006/mpev.1996.0069 8899721

[B22] Wen J Plunkett GM Mitchell AD Wagstaff SJ (2001) The evolution of Araliaceae: A phylogenetic analysis based on ITS sequences of nuclear ribosomal DNA. Systematic Botany 26: 144–167.

[B23] Xiang QB Lowry PL (2007) Araliaceae. In: Wu ZY Raven PH Hong DY (Eds) Flora of China. Beijing: Science Press & St.Louis: Missouri Botanic Garden Press 13: 435–491.

[B24] Yang D-Q (1981) The cyto-taxonomic studies on some species of *Panax* L. Zhiwu Fenlei Xuebao 19: 298–303.

[B25] Yoo K-O Malla KJ Wen J (2001) Chloroplast DNA variation of *Panax* (Araliaceae) in Nepal and its taxonomic implications. Brittonia 53(3): 447–453. 10.1007/BF02809800

[B26] Zhou J Huang WG Wu MZ Yang CR Feng KM Wu CY (1975) Triterpenoids from *Panax* Linn. and their relationship with taxonomy and geographical distribution. Acta Phytotaxomica Sinica 13(2): 29–45. [pls. 6–7]

[B27] Zuo Y-J Chen Z-J Kondo K Funamoto T Wen J Zhou S-L (2011) DNA barcoding of *Panax* species. Planta Medica 77(02): 182–187. 10.1055/s-0030-1250166 20803416

[B28] Zuo Y-J Wen J Ma J-S Zhou S-L (2015) Evolutionary radiation of the *Panaxbipinnatifidus* species complex (Araliaceae) in the Sino-Himalayan region of eastern Asia as inferred from AFLP analysis. Journal of Systematics and Evolution 15(3): 210–220. 10.1111/jse.12119

[B29] Zuo Y-J Wen J Zhou S-L (2017) Intercontinental and intracontinental biogeography of the eastern Asian – eastern North American disjunct *Panax* (the ginseng genus, Araliaceae), emphasizing its diversification processes in eastern Asia. Molecular Phylogenetics and Evolution 117: 60–74. 10.1016/j.ympev.2017.06.016 28743642

